# New York Academy of Medicine

**Published:** 1880-05

**Authors:** 


### NEW YORK ACADEMY OF MEDICINE.

Stated meeting, April 1, 1880, Fordyce Barker, M.D., LL.D., President, in the chair.

Dr. Frank H. Hamilton exhibited a new tonsillitome, with a few preliminary remarks as follows upon hypertrophy of the tonsils, and supplementary to Dr. Leffert's excellent paper read before the Academy:

From personal experience he regarded chronic hypertrophy of the tonsils as one of the manifestations of the strumous diathesis. He had scarcely seen it occur except in children who presented the general appearance seen in the condition called struma. He had noticed often that it was hereditary, and had known it to pass through several successive generations. He was not prepared to say that it generally had associated with it enlargement of the lymphatic glands in the neck. His impression was otherwise. Still, as he believed, that fact did not militate against the view that the condition belonged to the strumous diathesis. Again, the tonsils had a period of growth and decline. His impression was that the tonsils were seldom seen in early infants; they usually began to be seen at two, three, four or five years of age, and reached the climax of growth from the tenth to the twelfth year, the period during which application was made to the surgeon most frequently for relief, and it was rare to see hypertrophy of the tonsils after the twentieth year of age, at all events, anything that demanded extirpation. The question of the removal of the tonsils was not in dispute, but perhaps the circumstances under which their removal was proper was yet at issue. He had been in the habit of saying that, inasmuch as they had progress and decline, unless they occasioned serious inconvenience, there was no good reason why they should be removed. If, however, they were the seat of frequent attacks of acute inflammation, or if they interfered seriously with deglutition or respiration when the child was lying down, or if they were subject to acute attacks of neuralgia, or if they interfered with hearing, or in any way interfered with the eustachian tube, there was sufficient reason for their removal. With reference to hearing, it was well to consider whether the impairment was due to pressure produced by the enlarged tonsils, or to continuous inflammation producing actual thickening of the mucous membrane of the tube, and thus preventing audition. He had noticed that removal of the tonsils did not at once improve hearing; but that a period of one or two years must elapse before the benefit was experienced. Removal of the tonsils when actually inflamed was not to be practiced unless the patient was suffocating. Nor would he operate upon a person who was known to have a strong hemorrhagic diathesis, because of the liability to profuse hemorrhage. Dr. Hamilton then referred to three cases in which the hemorrhage was so considerable that it became alarming, all occuring in patients fourteen or fifteen years of age.

He then spoke of the different methods by which the tonsils have been extirpated, such as by the use of caustics, the ligature, and finally excision. He then described Velpeau's, Physic's, Warren's, Cox's, and Tiemann's tonsillotomes, and pointed out their defects. The instrument which he had employed, ever since beginning the practice of medicine and surgery, was a modification of Owen's tonsillotome, which he always suspected was really an instrument devised by that eminent surgeon, the late Dr. Alden March, of Albany.

The tonsil to be excised almost always hung downward. He had found it convenient to introduce the instrument into the mouth flatwise, with the face upward, then, after carrying it well back, turn it up edgeways, and in that manner the mouth was held open. He then passed his finger in, felt that the tonsil was properly engaged in the opening in the instrument, and when ready the guillotine was sent through the gland from *before backward.*

The President asked the indulgence of the Academy, while he referred to certain points in connection with chronic hypertrophy of the tonsils and its treatment. Inasmuch as no allusion had been made to one whose experience and extraordinary dexterity in manipulation had, perhaps, never been equalled, whatever may have been thought of his pathological views or practice, he would refer to the method of operating adopted by Dr. Horace Green, because he thought that all that could be should be known relative to the operation for the removal of the tonsils.

Dr. Green, after trying various methods, finally settled upon the following, which had been partially alluded to by Dr. Hamilton. His method was to seize the tonsil with a single hook, bring it forward, and then, with a bistoury, cut away the exact amount he wished to remove. He always operated upon the left tonsil first, standing in front of the patient, and cut from above downward. When the right tonsil was removed, he stood behind the patient, seized the gland, and cut from below upward. Scarcely ten seconds were required to perform the operation.

One fact which Dr. Green had noticed—and the President had made the same observation—was that the operation for removal of the right tonsil was the more painful of the two, and that there was more hemorrhage when the cut was made from below upward, than when made from above downward. Still, the removal of either tonsil was easily accomplished.

The President also believed the experience of all was, that in former times tonsils were often removed unnecessarily. He also believed that the tonsil had a period of increase, acme and decrease. He had seen tonsils become very large, and, in course of a few years, the hypertrophy had entirely disappeared without special treatment. Furthermore, there were some objections to the operation for removal, on account of the possible effect produced upon the voice. He thought removal was indicated not so much when the tonsils were simply hypertrophied as when there was disease of their interior structure, necessitating exsectoin in order to effect a cure.

Dr. A. C. Post remarked that he had been in the habit of using the instrument made by Mr. Tiemann, and had found that with it the tonsils could be removed with very great celerity, with ease to the surgeon, and but little discomfort to the patient.

Dr. A. B. Judson reported four cases of hip-joint disease, in the third stage, and presented three patients with the view to illustrate what could be accomplished by mechanical treatment of that disease. All were cases in which there was unquestionably complete destruction of the joint, and exaggerated evidence of general and local disturbance, and he thought that the results were sufficiently favorable to compare with the value claimed for exsection.

Case I.—A boy, seven years of age, had been diseased four years, when a long splint was adjusted without allowing the heel to reach the ground, and worn for one year. The constitutional disturbance was very severe, and cod liver oil, ferruginous tonics, etc., were administered, and nutrition sustained to the highest possible degree. From the subsidence of the local disturbance, at the end of a year, it became evident that fixation was no longer required, and the patient was furnished with another instrument which he wore for three years, and walked with crutches.

*Result.*—An active, healthy schoolboy. Stiffening of the joint, but fast walking was not prevented. Motion at the joint limited to slight flexion, abduction and adduction. Shortening half an inch distributed between the hip and knee. Limb smaller than its fellow. No evidence that the growth of the femur had been interrupted. Position of the femur that of slight flexion, but no abduction or adduction.

Case II.—Boy six years of age. Been under treatment eighteen months. Had worn short splint; been treated by means of weight and pulley, etc. Nine sinuses formed in the thigh. The long hip-splint was worn until it became evident that fixation was necessary.

a half. He had the history of hip joint disease occurring four years previously, and was supposed to have been cured. He had two or three discharging abscesses, and one opened after treatment had been begun. His recovery had been remarkably rapid under the influence of mechanical treatment, and his cure was complete. In 1871 he was greatly emaciated, and his thigh was flexed at about a right angle with the trunk. He had perfect motion in the joint. As Dr. Taylor's memory served him, there was no shortening. He had found only a few cases in which there was not more or less difference in the length of the limbs, but if there was an inch shortening with perfect motion in the joint, it would not be observable. The case pointed to the curability of hip-joint disease with mechanical treatment after suppuration had been established.

Case II.—A boy came under treatment in 1876 He was still wearing what Dr. Taylor called the median splint which was used particularly in cases in which a tendency to adduction was very great. The point of purchase in the median splint, a modification of the Davis's splint, was against the pelvis upon the opposite side. The boy was recovering with anchylosis. There was considerable flexion.

Case III.—A lad came under observation in 1877, in the third stage of hip-joint disease. A number of sinuses discharged at the time treatment was begun, but all had closed. He had a fair amount of motion, and was still wearing the supporting apparatus. Dr. Taylor regarded it as important to protect the joint for considerable time after extension became unnecessary, and the medial splint, or instrument, which the boy was wearing, was constructed for that purpose.

Case IV.—A boy, seven or eight years old, was, last summer, a fit subject for exsection of the hip-joint, and the case bore directly upon the question, as to whether or not a patient could be restored to fair health after being in a condition which would have justified excision. His general condition had so much improved that he was looking very well. When treatment was begun, his legs were strongly adducted, and pus was discharging from several sinuses.

All the sinuses except one had closed. He was wearing the long counter-extension splint on both legs, and the adjustment of the upper part was so made as to leave openings through which the discharge could pass without hindrance. His legs had been brought down parallel with each other, and the boy walked with crutches.

These cases were presented as average illustrations of what could be accomplished by mechanical treatment.

Dr. C. T. Poore thought that calling all cases of suppuration the third stage of hip-joint disease was an error, for suppuration might occur outside of the joint. Observers should, therefore, be more definite when speaking of the symptoms of the third stage.

With reference to exsection, he was unable to boast of any very good results. But from his cases he has thought several inferences might be drawn. He had excised the hip-joint thirteen times. *Four* of the patients were cured, and the remainder died. *One* died from phthisis, *four* from amyloid degeneration, *one* from cardiac thrombosis, *one* from tubercular miningitis, and *one* from exhaustion.

He thought the deaths from amyloid degeneration were due to constitutional causes. He had seen a patient with only a moderate amount of disease in the hip-joint, which soon showed evidences of amyloid degeneration of internal organs, and at autopsy, amyloid degeneration of the liver and kidneys was found. He had seen other cases which had had a duration of two or more years with profuse supperation, and the patient had died from pure exhaustion, and at autopsy no amyloid degeneration whatever was found. Why the degeneration appeared early in one case, and did not appear at all in another, even after prolonged supperation, he could not say, unless it was a constitutional condition. In all his cases of amyloid degeneration, there was a bad family history; and, in looking up cases of death from hip disease under treatment, he had been able to find in connection with amyloid degeneration a bad family history with regard to predisposition to tubercular disease.

He had collected forty-five cases of death from hip-joint disease not connected with excision. Of those, seven had tuberculosis, eight tubercular meningitis, sixteen amyloid degeneration, four were poor hospital cases, and ten died from exhaustion. These statistics showed that the danger of death from hip-joint disease was from constitutional disease, of course including the sixteen cases of amyloid degeneration as due to constitutional causes.

The treatment resolved itself into treating the predisposing causes. The cause being constitutional, the line of treatment to be followed in certain cases was readily determined.

With regard to family history, he thought that, in cases in which there was a bad history in that respect, the surgeon should guard as much as possible against profuse suppuration. With regard to results, what was desired was knowledge concerning those cases that passed from under notice. He believed that Mr. Holmes's remarks were true—namely, that one-third of the patients died, one-third were relieved somewhat, and one-third were cured. He thought all statistics pointed toward that conclusion.

With regard to excision, it must be remembered that in a vast majority of cases the patient had passed through the hands of gentlemen who did not believe in the operation, and finally they reach a surgeon who performs it as a last resort. He believed that if good results were obtained from excision, the operation must be performed early, and that then the patients would do better than to submit to long-continued mechanical treatment. It had been urged by those opposed to excisson that the patients died after the operation; but he claimed that they died, not from the operation, but despite excision and from intercurrent disease, due to too late surgical interference.

He wished that there was a society where the worst cases could be exhibited. Only a certain class of good cases were usually presented, but what surgeons wished to see was the worst cases.

He believed that when the joint had become disorganized better results could be obtained by excision than by mechanical treatment, but as good results as were wished had not been yet obtained. Of late he had given up excision, and tried sending his patients where they could get a change of climate and sea air, but had not seen any change in results. He thought that his mechanical treatment had been good.

There was one condition which he thought had not been touched upon sufficiently in connection with hip-joint disease, and that was extension of disease into the shaft of the bone, and remaining as chronic osteomyelitis. Of course those cases did badly.

He had seen one case of amyloid degeneration get well after excision. He thought that when the liver was enlarged from amyloid degeneration in connection with hip-joint disease, it was a case for excision rather than mechanical treatment.

Dr. L. A. Sayre remarked that he had nothing to add to what he had published many years ago. He knew of no alterations or amendments that should be made to the following doctrine, which he believed and taught: Medicine according to the actual demands of each case, and not for the purpose of combatting a theoretical morbific cause. See that the patient has sufficient food and that it is properly assimilated. Carefully study and regulate the hygienic surroundings of the patient. Give the parts involved in the disease absolute rest and freedom from pressure without materially interfering with the mobility of the joint. Extension in such a manner as to permit motion should be the object sought for in any mechanical appliance, except in cases where the inflamation is so violent that *absolute rest* is required for a time. In cases in which the bones and cartilages are involved, and there is suppuration, spontaneous recovery is extremely rare. In such cases nature can be aided by dilating sinuses leading to dead bone, perhaps making free openings in various directions, inserting drainage-tubes, etc., thus facilitating the ready and complete exit of the discharge. "If, notwithstanding this treatment, the discharge does not diminish, but rather increases; if the disease, instead of improving, progresses despite all your efforts to subdue it; if symptoms of progressive caries develop; if the general health of the patient is daily becoming undermined, and there are no symptoms indicating repair, the only justifiable treatment left for the surgeon is exsection of the joint. More can be done in half an hour with the knife and saw in way of removing dead bone than can be done by nature in many years."

Dr. Sayre then referred to the history of several cases, with photographs of the patients, in which there was much more motion than in any of the cases presented by Dr. Judson.

With regard to exsection, he had performed it in seventy-three cases and in no instance had the patient died in consequence of the operation. There was no immediate danger in connection with the operation. In cases in which the patient was suffering from constitutional disease, the earlier the operation was performed, if done at all, the better would be the result; and if nature did not show herself competent to remove the necrosed bone promptly, exsection should be resorted to rather than subject the patient to long-continued supperation and the dangers by which it was commonly accompanied.

Dr. J. C. Hutchison thought that it was imposible to fix the joint with any of the appliances exhibited. He maintained that it was impossible to fix the hip-joint except by apparatus extending to a considerable distance above upon the trunk. He also maintained that it was unnecessary to use any apparatus to produce fixation, because when the joint was diseased, it was firmly fixed by the constant action of the peri-articular muscles. He also maintained that it was unnecessary to use any apparatus to produce extension. He had not yet seen any apparatus that did not require constant attendance to enable it to keep up extension. But, even if extension could be made by any of the apparatus used for that purpose, he maintained that it was not necessary, for sufficient extension could be secured by the weight of the limb. [See Medical Record, vol. xv., No. 19.] A greater amount of extension could be obtained by putting a high heel and sole on the shoe worn on the well side, and giving the patient a pair of crutches, than in any other way. He believed it to be impossible to separate the head of the bone from the acetabulum by any mechanical appliance, and all an apparatus could do was to prevent the head of the bone from jamming up into the acetabulum, and thus prevent pain.

